# Life satisfaction and job and personal resources among public workers with non-standard work schedules

**DOI:** 10.1186/s12889-024-18575-x

**Published:** 2024-04-23

**Authors:** Jennifer M. Cavallari, Sierra M. Trudel, Megan N. Miskovsky, Rick A. Laguerre, Alicia G. Dugan

**Affiliations:** 1 UConn School of Medicine, Farmington, CT USA; 2 University of Connecticut, Storrs, CT USA; 3 Montclair State University, Montclair, NJ USA; 4 Department of Public Health Sciences, UConn School of Medicine, 263 Farmington Avenue, MC 6325, 06030-6325 Farmington, CT USA

**Keywords:** Life satisfaction, Well-being, Worker, Job resources, Personal resources

## Abstract

**Background:**

While the link between non-standard work schedules and poor health outcomes is established, few studies have examined how resources both in and outside of work can support the well-being of workers with non-standard work schedules.

**Methods:**

Using a cross-sectional survey, we assessed the association between one facet of well-being, life satisfaction, and job and personal resources. In 2019, an electronic survey was administered to two unionized, public service populations who work non-standard work schedules: transportation maintainers and correctional supervisors. We assessed life satisfaction with a 10-item scale; a broad set of job resources (reward satisfaction, supervisor support, co-worker support, schedule satisfaction, and working hours fit); and a broad set of personal resources (health status, sleep, physical activity, and finances). We used log-binomial regression models to estimate prevalence ratios and 95% confidence intervals among statistically significant univariate predictors.

**Results:**

Of the 316 workers surveyed, the majority were male (86%), White (68%), and reported positive life satisfaction (56%). In multivariate models, the prevalence of positive life satisfaction was higher in workers reporting reward satisfaction (PR:1.35, 95% CI: 1.11, 1.65; *p* = 0.003), good work schedule fit (PR:1.43, 95% CI: 1.12, 1.83; *p* = 0.004), good health (PR:2.92, 95% CI: 1.70, 4.99; *p* < 0.0001), and good finances (PR:1.32, 95% CI: 1.01, 1.72; *p* = 0.04).

**Conclusion:**

Employers should consider increasing work recognition, as well as improving schedule fit, financial well-being, and overall good health in support of worker life satisfaction and ultimately well-being.

**Supplementary Information:**

The online version contains supplementary material available at 10.1186/s12889-024-18575-x.

## Background

Public service and maintenance workers are often required to work outside the standard workday or week. While the adverse impact of non-standard work schedules (i.e. long work hours, evening work, and weekend work) on worker well-being is established [1–3], few studies have demonstrated how to support workers whose jobs require non-standard work schedules.

Within the field of occupational health, there has been an expansion to understand both the predictors of injury and illness as well as the factors influencing well-being [4]. Importantly, there is a need to identify the modifiable factors that improve worker well-being to inform programs that increase worker and business outcomes [5]. 

Although no standard definition for well-being exists [6], one way of understanding well-being is through the components of subjective well-being. Subjective well-being can be classified within three sub-domains; hedonic well-being or experiences of positive feelings or happiness; eudaimonic well-being or feeling a sense of purpose or meaning; and evaluative well-being or level of satisfaction with one’s life as a whole. Numerous definitions of well-being include life satisfaction as a prominent dimension both broadly [7–9], as well as for workers specifically [10]. 

Like well-being, no single definition of life satisfaction exists, rather our understanding of life satisfaction is guided by two theories, the *top-down* approach which attributes life satisfaction to stable personal characteristics such as personality, and the *bottom-up* approach which attributes life satisfaction to contentment across multiple domains [11]. The bottom-up approach posits that as needs across the life domains are satisfied, life satisfaction increases. Life satisfaction is informed by satisfaction within the area or domains of: marriage/significant relationships, physical health, family, neighborhood/housing, friendships, education, leisure activities, and emotional health [12]. The bottom-up approach is often used to contextualize the job and work characteristics that relate to life satisfaction [11]. 

Work satisfaction is a component of life satisfaction since a person appraises the time they spend working when thinking about the quality of their overall life, which is an appraisal of activities both in and outside of work [13]. Work and life satisfaction are reciprocally related and they influence each other over time, meaning that over the course of several years, when people are satisfied with their jobs, they are likely to be happier with life, and vice versa [14]. The reason for this is because people spend a significant time at work, and positive/negative events at work can impact the amount of time and energy people have to commit towards their personal lives [15]. When people have a vibrant and meaningful life outside of work, they have more energy to commit to work. Thus, although work and life satisfaction are related, they are distinct in that they uniquely contribute to performance at work [16]. Therefore, evaluating life satisfaction with respect to worker well-being has added value for ensuring workers can meet the demands of their jobs while ensuring good mental health.

Work and non-work activities both contribute to the degree that a person has time, energy, and motivation to develop their personal life [17], contributing to overall life satisfaction [18]. There is a growing recognition that the impact of work domains on life satisfaction, independent of job satisfaction, deserves additional investigation [11]. Erdogan et al. [11] argue that since job satisfaction only partially predicts satisfaction at work and since some aspects of work impact non-work domains, for example through the spillover of work stress into family relationships, there is a need to further understand how life satisfaction relates to organizational factors, specifically for under-researched groups such as workers with non-standard work schedules.

The Job Demands-Resources (JD-R) model can be used to contextualize workplace factors that impact worker well-being [19]. The model defines job demands as aspects of a person’s job that require the expenditure of physical and psychological effort. When workers exert high effort to meet job demands, stress may occur when these demands exceed or deplete their resources. Job resources have been described as “those physical, psychological, social or organizational aspects of the job that are either/or: 1) functional in achieving work goals; 2) reduce job demands and the associated physiological and psychological costs; and 3) stimulate personal growth, learning, and development” [20]. Rather than thinking of job demands and resources in a narrow or specific way, the JD-R model can be approached as a framework to broadly explore demands and resources that impact well-being [21]. For example, the JD-R model can be extended beyond job-related resources to also include personal resources [22]. Personal resources include material objects (e.g., a home), advantageous conditions (e.g., being employed), individual characteristics (e.g., self-efficacy), and instrumental resources that can be used to acquire other resources (e.g., money) [23]. In addition to countering the adverse impact of job demands, resources play an important and independent role in worker well-being [20]. Abundant job resources can fuel a motivational process that leads to work engagement and other positive outcomes such as subjective well-being [22]. The impacts of the COVID-19 pandemic shed light on the interaction of job and personal resources in predicting worker well-being outcomes, where resources in the personal domain such as family support enriched resources from the organization such as organizational health climate [24].

As part of the WorkTime study we sought to explore the association between resources and one facet of well-being, life satisfaction, within a cohort of blue-collar workers with non-standard work schedules (including long and/or irregular work hours). This is an extension of prior research applying the JD-R model to examining predictors of life satisfaction in nurses [25, 26] and teachers and social workers [27]. In line with the US Surgeon General’s recommendation [5], we sought to identify workplace factors that support worker life satisfaction as a component of well-being. Importantly, we were interested in the role of both job and personal resources. Regarding job resources, we focused on social support (from both co-workers and supervisors) and recognition, as well as resources in the form of satisfaction with aspects of working time. In terms of personal resources, we focused on health, health behaviors (sleep and physical activity), as well as finances which are facets of worker well-being widely impacted by work itself [10]. We hypothesized that both job and personal resources have a positive and independent association with life satisfaction for workers with non-standard work schedules.

## Methods

### Study design and data collection

The current secondary data analysis uses a subset of data from the WorkTime study, a cross-sectional, mixed methods community-based participatory research study of blue-collar workers examining the associations between working time characteristics and worker and family health and well-being, as described in more detail elsewhere [1, 28–30]. The study population within this analysis includes workers with non-standard schedules employed by a New England state as correctional supervisors or transportation maintenance garage workers. Briefly, unionized correctional supervisors were recruited to take the survey in the Spring of 2019 before a mandatory mental health training. Transportation garage employees were recruited to take the survey in the Summer of 2019, at the maintenance garage where they worked before a mandatory hearing conservation training. All surveys were completed on work time with company and union approval. The procedures performed in the study were reviewed and approved by the UConn Health Institutional Review Board. Informed consent was obtained by all participants enrolled in the study.

### Variables and measures

We used a participatory survey design approach where worker representatives were asked to provide feedback on survey items, as previously performed by the researchers in the correctional supervisor population [31].

#### Life satisfaction

Consistent with the *bottom-up* approach [11], the outcome measure, *life satisfaction* was assessed as a summary of satisfaction across broad domains. The original scale asked participants to indicate how satisfied they felt across 10 domains (marriage or other significant relationship, health, family life, neighborhood, sex life, housing, friendships, education, standard of living, leisure activities) on a 5-point Likert scale ranging from 1 = very dissatisfied to 5 = very satisfied. With feedback from the worker supervisors, the sex life item was removed, which is consistent with a prior study in a worker population [32]. Furthermore, the single ‘health’ item was adapted into 2 items, ‘physical health’ and ‘emotional health'. A composite score of life satisfaction was calculated by averaging each rating across the 10 items (with no more than 2 missing items), which had a Cronbach’s α of 0.90. A dichotomous variable indicating positive life satisfaction was created from the summary measure, with a cut-off of 4 (somewhat satisfied) or higher (very satisfied).

#### Job and personal resources

Job and personal resource items were selected from existing WorkTime survey items as guided by the JD-R model. Survey measures including citations are provided in Supplemental Table 1. Job resources were characterized across two areas including social resources (reward satisfaction, supervisor support, co-worker support) and job resources (schedule satisfaction, schedule control satisfaction, working hour fit).

##### Job resources

We included 2-items to assess *reward satisfaction* within the domains of recognition and appreciation. Both reward satisfaction items were adapted from items from the Job Satisfaction Survey [33]. The two items were averaged and dichotomized based on a cut-point of 4 or higher to indicate reward satisfaction. *Supervisor and co-worker social support* were assessed using survey items from the job content questionnaire based on the job-strain model [34]. For each area of support, the two items were separately averaged to create summary scales for supervisor and co-worker support which were then dichotomized using a cut-point of 3 or higher.

*Schedule satisfaction* items included an adapted item of general schedule satisfaction [35] and a new item developed by the researchers on work hour satisfaction. The two items were averaged and dichotomized based on a cut-point of 4 or higher to indicate schedule satisfaction. *Satisfaction with schedule control* was assessed with two items adapted from a prior survey [36] and dichotomized based on a cut-point of 4 or higher to indicate schedule control satisfaction. The *fit of working hours* with personal life was assessed with 1-item, which was dichotomized to a score of 3 or higher.

##### Personal resources

We assessed four personal resources through single items for health, sufficient sleep, physical activity, and finances. *Health* was assessed with one item of the SF-12 [37]. A cut-point of 3 or higher was used to identify “good health”. *Sleep* was assessed with an adapted item from the Pittsburgh Sleep Quality Index [38] and dichotomized based on a cut-point of 7 h or higher to indicate sufficient hours of sleep. The *physical activity* variable was dichotomized based on a cut-point of 4 or higher indicating often or always meeting the weekly physical activity goal of the 2008 US Physical Activity Guidelines [39]. The *financial situation* original variable was dichotomized based on a cut-point of 2 or lower indicating financial comfortability of meeting basic needs with some money left over for extras or the ability to live comfortably.

#### Additional demographic and occupational factors

We assessed additional demographic and occupational factors including gender, age, race, marital status, responsibility for children, tenure, and whether or not the respondent worked more than one job. The frequency of non-standard work schedule characteristics (long work hours, overnight work, unexpectedly working when not scheduled, weekend work) were assessed on a 5-point Likert scale with response options ranging from never to always [30], and dichotomized at a cut-point of 3 (sometimes) or higher.

### Data analysis

We examined the frequency of the demographic and occupational characteristics by life satisfaction status. We examined univariate associations between the dichotomized life satisfaction measure and sociodemographic and occupational characteristics using ANOVA. This same approach was used to examine the univariate associations between life satisfaction and job and personal resources. In addition, we calculated the prevalence to examine the association between each individual resource and positive life satisfaction. Statistically significant (*p* < 0.05) predictors were considered candidates for multivariate models using log-binomial regression to estimate prevalence ratios and 95% confidence intervals. The correlations between measures were assessed using Pearson correlation coefficients. We used mixed effects models to assess whether it was necessary to account for clustering within company by evaluating the intra-class correlation coefficients (ICC). The ICC was low at 0.002, indicating that only 0.2% of the variance in life satisfaction is accounted for by the company, indicating that mixed models were not necessary.

To assess the independent and combined effects of job and personal resources, models were built and examined stepwise. First, the influence of job resources was examined (Model 1) and next, the influence of personal resources (Model 2). Multivariate models examining the influence of both job and personal resources simultaneously (Model 3) were built including statistically significant predictors from the prior Models 1 and 2. Beta coefficients from the regression models were transformed into prevalence ratios that compare the prevalence of life satisfaction between the low and high values of each dichotomized predictor. Statistical analysis was completed in SAS (version 9.4, Cary NC) using the Proc Genmod function. The model fit was assessed using Akaike information criterion (AIC).

## Results

A total of 318 workers were surveyed with 316 completing the life satisfaction measure. A majority of surveyed workers were male (86%), over 45 years (44%), White (68%), married or partnered (70%), and had responsibility for children (60%) (Table 1). A little over half were employed by the Department of Transportation (*n* = 173) and the remainder (*n* = 143) by the Department of Correction (Table 1) with the majority (75%) having 5 or more years of job tenure. The majority (*n* = 286; 93%) of respondents reported sometimes or more often experiencing one or more features of non-standard work schedules including long (> 48) weekly hours (76%), overnight work (62%), working unexpectedly (60%), or working on weekends (80%). Positive life satisfaction was observed among 56% (*n* = 176) of workers. We observed no statistically significant differences in life satisfaction by selected demographic or occupational characteristics (Table 1).

**Table 1:** Demographic and occupational characteristics by life satisfaction status

	All *n* (%)		Positive *n* (%)		Neutral/Neg *n* (%)		*p*-value^a^
Male	268	(86)	150	(87)	118	(84)	0.46
Age (years)							
Under 35	71	(22)	44	(25)	27	(19)	0.59
36 to 45	108	(34)	55	(31)	53	(38)	
Over 45	137	(43)	77	(44)	60	(43)	
Race							0.69
People of color, multi-racial	97	(37)	52	(31)	45	(33)	
White	205	(68)	115	(69)	90	(67)	
Marriage Status							0.07
Married or partnered	219	(70)	128	(74)	91	(65)	
Single	92	(30)	44	(26)	48	(35)	
Has child(ren) responsibility	184	(60)	102	(60)	82	(59)	0.97
Employer							0.41
Dept of Corrections	143	(45)	76	(43)	67	(48)	
Dept of Transportation	173	(55)	100	(57)	73	(52)	
Tenure, years							0.14
Under 5	78	(25)	37	(21)	41	(30)	
5 to 15	135	(43)	78	(45)	57	(41)	
Over 15	100	(32)	59	(34)	41	(30)	
Works more than one job	106	(34)	57	(33)	49	(35)	0.70

^a^ANOVA was used to assess differences in life satisfaction

The distributions of both job and personal resources are summarized in Table 2. The majority of respondents were neutral or dissatisfied with rewards (60%), yet agreed that they felt social support from their co-workers (68%) and supervisors (72%). The majority of respondents were neutral or dissatisfied with schedule control (87%), yet satisfied with their schedule (58%) and had a very well or well working hour fit (69%). With the exception of schedule control satisfaction, respondents indicating higher job resources had 1.56–1.82 times the prevalence of positive life satisfaction compared to respondents indicating lower job resources. Likewise, each of the job resources, with the exception of schedule control satisfaction, had a statistically significant difference by life satisfaction status with higher distributions of job resources among respondents with positive life satisfaction. The majority of job resources were weakly, positively correlated with each other (0.01 to 0.36), with the exception of reward and schedule satisfaction which was moderately correlated (0.50) (Supplemental Table 2).

**Table 2:** Distribution of work and personal resources by life satisfaction status

	All *n* (%)		Positive *n* (%)		Neut/Neg *n* (%)		PR	*p*-value^a^
**Job Resources**								
Reward satisfaction							1.82	<0.0001
Satisfied	124	(40)	95	(55)	28	(21)		
Neutral/Dissatisfied	188	(60)	79	(45)	109	(79)		
Co-Worker social support							1.79	<0.0001
Agree	214	(68)	139	(79)	75	(54)		
Disagree	99	(32)	36	(21)	63	(46)		
Supervisor social support							1.60	0.0002
Agree	226	(72)	141	(81)	85	(62)		
Disagree	87	(28)	34	(19)	53	(38)		
Schedule satisfaction							1.56	<0.0001
Satisfied	180	(58)	118	(68)	62	(45)		
Neutral/Dissatisfied	131	(42)	55	(32)	76	(55)		
Schedule control satisfaction							1.06	0.69
Satisfied	41	(13)	24	(14)	17	(12)		
Neutral/Dissatisfied	270	(87)	149	(86)	121	(88)		
Working hour fit							1.60	< 0.0001
Very well/Well	217	(69)	137	(78)	80	(57)		
Not very well/Not well at all	99	(31)	39	(22)	60	(43)		
**Personal Resources**								
Health							3.35	<0.0001
Good or better	255	(81)	162	(94)	93	(66)		
Fair or poor	58	(19)	11	(6)	47	(34)		
Sleep							1.35	0.003
7 or more hours	112	(35)	75	(43)	37	(26)		
Less than 7 hours	204	(65)	101	(57)	103	(74)		
Physical activity							1.33	0.005
Often or always	118	(38)	78	(45)	40	(29)		
Half the time, rarely or never	195	(62)	97	(55)	98	(71)		
Financial situation							1.60	0.0002
Meet or exceed basic expenses	233	(74)	144	(82)	89	(64)		
Just meet or don’t have enough to meet basic expenses	83	(26)	32	(18)	51	(36)		

PR = prevalence ratio. ^a^ANOVA was used to assess differences in life satisfaction

In terms of personal resources, the majority of respondents reported good or better health (81%), and a financial situation where they could meet or exceed basic expenses (74%), yet only 35% of respondents reported 7 or more hours of sleep on average and 38% of respondents often or always met physical activity guidelines. Respondents indicating good or better health had 3.35 the prevalence of positive life satisfaction as compared to respondents indicating fair or poor health. Respondents indicating personal resources of sufficient sleep, frequent physical activity and good finances had 1.33–1.60 times the prevalence of positive life satisfaction as compared to respondents with low or deficient personal resources. Each of the personal resources had a statistically significant difference by life satisfaction with a higher distribution of the personal resources of health, sleep, and financial situation among positive life satisfaction respondents, yet not physical activity. The personal resource variables were very weakly correlated with each other (0.02 to 0.20) as well as with the job resource variables (0.02 to 0.16) (Supplemental Table 2).

Next, we examined how the prevalence of life satisfaction varied by job and personal resources both independently (Table 3, Models 1 and 2) and combined (Table 3, Model 3). A statistically significant higher prevalence of life satisfaction was observed among participants indicating higher reward satisfaction (PR 1.47, 95% CI: 1.10, 1.95; *p* = 0.01), co-worker social support (PR 1.38, 95% CI: 1.01, 1.89; *p* = 0.04), and good work hour fit (PR 1.34, 95% CI: 1.02, 1.76; *p* = 0.03) (Table 3, Model 1). In terms of personal resources, a higher prevalence of life satisfaction was observed among participants reporting good health (PR 3.05, 95% CI: 1.77, 5.24; *p* < 0.0001) and good finances (PR 1.46, 95% CI: 1.10, 1.94; *p* = 0.01), Table 3, Model 2. Considering both job and personal resources together, the prevalence of life satisfaction remained statistically significant and higher in workers reporting reward satisfaction (PR:1.35, 95% CI: 1.11, 1.65; *p* = 0.003), good work schedule fit (PR:1.43, 95% CI: 1.12, 1.83; *p* = 0.004), good health (PR:2.92, 95% CI: 1.70, 4.99; *p* < 0.001), and good finances (PR:1.32, 95% CI: 1.01, 1.72; *p* = 0.04). While a higher prevalence of life satisfaction was observed among those reporting good co-worker social support, the association was no longer statistically significant in the full model. The model fit improved from Model 1 and Model 2 to Model 3 which had the best fit with an AIC of 347.

**Table 3:** Association between positive life satisfaction by work and personal resources

	Model 1				Model 2				Model 3			
	PR	95% CI		*p*-value	PR	95% CI		*p*-value	PR	95% CI		*p*-value
Job Resources												
Reward satisfaction	1.47	1.10	1.95	0.01					1.35	1.11	1.65	0.003
Co-worker social support	1.38	1.01	1.89	0.04					1.20	0.91	1.59	0.19
Supervisor social support	1.07	0.78	1.48	0.67								
Schedule satisfaction	1.02	0.75	1.39	0.92								
Working hour fit	1.34	1.02	1.76	0.03					1.43	1.12	1.83	0.004
Personal Resources												
Health					3.05	1.77	5.24	<0.0001	2.92	1.70	4.99	< 0.0001
Sleep					1.17	0.98	1.39	0.09				
Physical activity					1.10	0.93	1.32	0.27				
Financial situation					1.46	1.10	1.94	0.01	1.32	1.01	1.72	0.04
Model Fit Diagnostics												
AIC	388				379				347			

## Discussion

Among public service and maintenance workers with non-standard work schedules working in transportation and corrections, approximately half indicated positive life satisfaction. We observed independent, positive associations between job and personal resources and life satisfaction. This adds to the growing research evaluating the independent role of job resources in worker well-being [40, 41]. A novel finding of the current study is that the association between life satisfaction and both reward satisfaction and work schedule fit persisted after the adjustment for good health and financial status. This suggests a robust and independent association between job resources, specifically reward and work schedule fit, and life satisfaction. This is especially relevant given the work by Mauno et al. [42] indicating that job resources are better predictors of employee engagement than job demands.

We observed that workers indicating high reward satisfaction and good work schedule fit also had a higher prevalence of life satisfaction. Our assessment of reward satisfaction included measures of satisfaction related to recognition and appreciation. Prior research has demonstrated the role reward satisfaction plays in work related factors such as turnover intentions [43] and job satisfaction [44], yet it is unclear how reward satisfaction impacts worker well-being for those working non-standard work schedules. Interestingly, reward satisfaction remained positively associated with life satisfaction even after adjusting for financial situation. This may suggest that recognition and appreciation is more than just monetary compensation but rather encompasses the acknowledgement of a job well done. This is consistent with the work done by Ray [45] who also found an association between job satisfaction and well-being after controlling for financial status with job satisfaction accounting for a 14% higher current life evaluation score.

Workers reporting good work schedule fit also indicated a higher prevalence of life satisfaction even after accounting for reward satisfaction and personal resource factors. Work schedule fit broadly encompasses workers’ satisfaction with how well one’s work hours fit with their non-work life and enable them to participate in family and social life. On one hand, literature around work-life/family conflict suggest that the demands of one domain (work or life/family) when incompatible can deplete the resources and success in the other [46]. On the other hand, the theory of work-life enrichment, suggests that the two domains can also provide enrichment or additional resources for one and other, such that one’s work life can bolster one’s non-work life and vice versa [47]. Studies exploring non-standard work schedules have found associations of shift work schedules with decreased leisure time and low social participation suggesting engagement in shift work limits the worker’s ability to engage in community or non-work activities which may otherwise bolster personal resources [48]. In accordance with the JD-R model, work schedule fit can be considered a personal resource that may help mitigate the negative effects of work. Should a worker’s non-work life complement a non-standard work schedule, the strain on the worker may be reduced and their well-being would be higher because they would be receiving fulfillment from both the job and life domains with limited competing demands.

Both supervisor and co-worker social support showed statistically significant associations with life satisfaction in univariate associations. Supervisor support had a significant positive univariate association with job satisfaction which was non-significant when job resources and demands were introduced as predictors in a regression model. This suggests that the direct impact of supervisor support may be diminished in the presence of abundant resources. This is consistent with research indicating that leadership’s impact on job satisfaction and ultimately well-being is through their ability to shape the work environment through job resources and demands [40]. Co-worker support, while statistically significant in the model with additional job resources was attenuated when adding personal resources including good health and finances. In looking at the relationship between co-worker support and physical health specifically, social connectedness has been shown to play an integral role in health behaviors and longevity [49, 50]. 

In terms of personal resources, while each of the four personal resources were associated with a higher prevalence of life satisfaction in the univariate associations, only health and good finances, yet not the specific health behaviors of adequate sleep or exercise remained statistically significant in the multivariate models. Health and finances have been shown to demonstrate a reciprocal relationship with increased financial stress being correlated with increased physical illness [51], such as sleep loss, compromised immune system, and heart disease [52]. 

Recognition and appreciation at work is a job resource that satisfies esteem needs [53]. Another job resource, work schedule fit, is instrumental in enabling workers to participate in family or social life outside of work, meeting important relational and belonging needs. This may be especially relevant among workers with non-standard work schedules. Good health status and a comfortable financial situation - which we characterize as personal resources - are nonetheless affected by work and meet basic physiological and security needs. Research on need fulfillment at work specifically identifies health/safety needs and economic family needs as being among the major needs that workplace experiences can fulfill for workers, contributing to their job satisfaction and ultimately life satisfaction [53]. Similar research has shown that decent work conditions (i.e., those that are safe, offer healthcare benefits, provide adequate compensation, permit free time for non-work activities, and are aligned with personal values) are associated with job satisfaction and well-being [54]. Findings suggest that employers can enhance life satisfaction among their workers by providing resources that enable a good financial situation, good health, high feelings of esteem through recognition and appreciation, and work schedules that allow workers to fully engage in their social and family life outside of work. Future research on associations between resources and well-being should more fully consider the role of need satisfaction as an explanatory factor.

The study results must be balanced with respect to the study limitations. In considering the generalizability of our findings, it is important to acknowledge that this unionized population of workers have access to good pay, health insurance, well-being programs, as well as job stability, all are important predictors of life satisfaction [55] and well-being more broadly [10]. The impact of job and personal resources in the absence of these benefits should be further examined. Job satisfaction has consistently shown strong correlations with life satisfaction, and specifically financial satisfaction in combination with job satisfaction has been shown to elicit higher life satisfaction overall [13]. Future studies should further explore the facets of job satisfaction with regard to life satisfaction. Likewise, the majority of the population was male and White and given the inequities in occupational health outcomes and exposures and the intersection with the social determinants of health [56], additional studies should also examine the role of resources in life satisfaction among more diverse groups and how these intersect with social contexts.

Furthermore, our sample size was small and may have limited our ability to detect positive associations. To maximize study power, we present a combined analysis of correctional supervisors and transportation maintainers which are similar in demographics yet disparate in work tasks. In univariate associations, company was not a predictor of life satisfaction and in a sensitivity analysis (data not shown) adjustment for company along with gender and age had minor changes in effect estimates that did not impact interpretation of the results. For each of the work and personal resources that had a statistically significant univariate association with life satisfaction (Table 2), the prevalence ratio was reduced when moving from univariate to multivariate models. While the low correlation between these variables justify their inclusion in multivariate models, small sample size may have limited the ability to detect statistically significant associations and the role of these resources including social support, sleep, and exercise in life satisfaction should be further explored.

While we were able to examine how job and personal resources impact worker well-being, it should be noted that we only examined a small number of resources. Additional studies may wish to examine the role of job resources more comprehensively including facets of organizational resources (e.g. communication [57], justice and fair pay [58],, trust in leadership), employee development resources [59](e.g. career perspectives, mentorship programs, networking opportunities, professional development), as well as more fully explore job resources [58] (e.g. task variety, use of skills, autonomy, participation in decision making) [22]. For example, autonomy in one’s job may be partially captured in the variable schedule control, but autonomy in one’s job goes beyond schedule making and it would be important to capture levels of autonomy beyond that, such as within the tasks employees are expected to perform. Similarly, the social resources used in this study focus solely on support and acknowledgement from co-workers and leadership, future studies may wish to incorporate additional social resources such as social support from both friends and family as these may influence work schedule fit and satisfaction. Likewise, a further examination of the role of job demands may elucidate drivers of worker well-being as there is evidence that job stressors (time pressures, concentration demands, work organization problems, and uncertainty) predict future worker well-being [60]. Likewise, we dichotomized resources into high/low categories. Future studies should attempt to quantify an ideal level of resources that is most conducive to worker success and well-being. Lastly, we only examined one facet of well-being, life satisfaction and there may be benefit in looking at additional dimensions of well-being to further elucidate important determinants.

The results of this study can inform workplace initiatives to support worker well-being. While there is a strong evidence base for the effectiveness of individual-level psychological interventions for improving worker well-being [61], there is a call to explore multi-level interventions that include organizational as well as individual-level components when addressing mental health [62] that may also be relevant to life satisfaction and broader well-being. There is value for workers and workplaces in supporting worker well-being [5] through prioritization worker health through the protection from workplace hazards as well as through healthcare benefits and programs that support health behaviors. There is growing evidence and examples for taking a holistic approach to worker well-being focusing both on protection from work-related hazards and promotion of worker well-being [5, 63]. In line with prior research [64], our study indicates that employees benefit from increased organizational resources including worker recognition programs, flexible work arrangements, and programs that support worker financial health.

## Conclusion

Both job and personal resources contribute to worker life satisfaction. Workplaces can consider increasing work recognition, as well improving worker schedule fit, financial well-being, and overall good health in support of worker life satisfaction and ultimately well-being.

### Electronic Supplementary Material

Below is the link to the electronic supplementary material.

Supplementary Material 1

Supplementary Material 2

## Data Availability

The dataset generated and analyzed during the study is available from the corresponding author, [JMC], upon reasonable request.

## Abbreviations

CI: Confidence Interval
JD-R: Job Demands Resources
PR: Prevalence Ratio
SD: Standard Deviation
