# Supplementary material for: Life satisfaction and job and personal resources among public workers with non-standard work schedules
Source: BMC Public Health. 2024 Apr 23;24:1133. doi: 10.1186/s12889-024-18575-x (PMC11040770; doi:10.1186/s12889-024-18575-x)
Supplement: Supplementary file 1 — Supplementary Material 1 [file 12889_2024_18575_MOESM1_ESM.docx]

**Supplemental Table 1: Selected Job and Personal Resource Survey Items**

| **Construct** | **Variable (source)** | **Item** | **Response Options** | **Alpha** |
| --- | --- | --- | --- | --- |
| **Job Resources** | | | | |
|  | Reward Satisfaction (Adapted from [33]) | I am satisfied with the recognition I receive when I do a good job at work  I am satisfied with the appreciation I feel for the work that I do | 5-point Likert scale from 1 (Strongly Disagree) to 5 (Strongly Agree) | 0.93 |
|  | Supervisor Support [34] | My supervisor pays attention to what I am saying.  My supervisor is helpful in getting the job done. | 4-point Likert scale from 1 (Strongly Disagree) to 4 (Strongly Agree) | 0.91 |
|  | Co-worker Support [34] | People I work with take a personal interest in me.  People I work with are helpful in getting the job done. | 4-point Likert scale from 1 (Strongly Disagree) to 4 (Strongly Agree) | 0.62 |
|  | Schedule Satisfaction (adapted from [35]) | I am satisfied with my work schedule.  I am satisfied with the number of hours I work. | 5-point Likert scale from 1 (Strongly Disagree) to 5 (Strongly Agree) | 0.99 |
|  | Schedule Control (adapted from [36]) | I would like more control over my work schedule  I would like my work schedule to be more predictable | 5-point Likert scale from 1 (Strongly Disagree) to 5 (Strongly Agree) | 0.67 |
|  | Working Hour Fit (original item) | In general, how do your working hours fit in with your family or social commitments outside work?) | 4-point scale (1=very well, 2=well, 3-not very well; 4= not well at all) | N/A |
| **Personal Resources** | | | | |
|  | Health [37] | In general, would you say your health is... | 5-point scale (1=Poor; 2=Fair; 3=Good; 4=Very Good; 5=Excellent) | N/A |
|  | Sufficient Sleep (adapted from [38]) | In the past month, about how many hours of sleep did you typically get per 24-hour period during the work week? | Response option from 0 to >10 hours in hourly increments | N/A |
|  | Physical Activity (adapted from [39]) | Health experts say that you should do strength training exercise twice a week plus do other activities that increase your heart rate and breathing on several days each week. How often do you meet this goal? | 5-point scale (1=never; 2=rarely; 3=half the time; 4=often; 5=always) | N/A |
|  | Finances (original item) | How would you describe your current household financial situation? | 4-point scale (1 = able to live comfortably; 2 = meet basic expenses with a little left over for extras; 3 = just meet basic expenses; 4 = don't even have enough to meet basic expenses) | N/A |
