# Supplementary material for: Life satisfaction and job and personal resources among public workers with non-standard work schedules
Source: BMC Public Health. 2024 Apr 23;24:1133. doi: 10.1186/s12889-024-18575-x (PMC11040770; doi:10.1186/s12889-024-18575-x)
Supplement: Supplementary file 2 — Supplementary Material 2 [file 12889_2024_18575_MOESM2_ESM.docx]

**Supplemental Table 2: Summary statistics and correlations between work and personal resources**

|  |  | **n** | **Mean** | **(SD)** | **1** | **2** | **3** | **4** | **5** | **6** | **7** | **8** | **9** |
| --- | --- | --- | --- | --- | --- | --- | --- | --- | --- | --- | --- | --- | --- |
| **1** | Reward satisfaction | 312 | 0.40 | (0.49) | 1.00 |  |  |  |  |  |  |  |  |
| **2** | Co-worker social support | 313 | 0.68 | (0.47) | 0.36 | 1.00 |  |  |  |  |  |  |  |
| **3** | Supervisor social support | 313 | 0.72 | (0.45) | 0.36 | 0.33 | 1.00 |  |  |  |  |  |  |
| **4** | Schedule satisfaction | 311 | 0.58 | (0.49) | 0.50 | 0.21 | 0.19 | 1.00 |  |  |  |  |  |
| **5** | Schedule control satisfaction | 311 | 0.13 | (0.34) | 0.11 | 0.10 | 0.01 | 0.06 | 1.00 |  |  |  |  |
| **6** | Working hour fit | 316 | 0.69 | (0.46) | 0.20 | 0.11 | 0.13 | 0.28 | 0.12 | 1.00 |  |  |  |
| **7** | Health | 313 | 0.81 | (0.39) | 0.14 | 0.10 | 0.11 | 0.03 | -0.13 | -0.02 | 1.00 |  |  |
| **8** | Sleep | 316 | 0.35 | (0.48) | 0.15 | 0.03 | 0.12 | 0.09 | 0.03 | 0.16 | 0.12 | 1.00 |  |
| **9** | Physical activity | 313 | 0.38 | (0.49) | 0.10 | 0.10 | -0.02 | 0.10 | 0.03 | 0.09 | 0.20 | 0.02 | 1.00 |
| **10** | Financial situation | 316 | 0.74 | (0.44) | 0.16 | 0.20 | 0.08 | 0.16 | -0.02 | 0.03 | 0.13 | 0.10 | 0.004 |
